# Influence of Polysaccharides’ Molecular Structure on the Antibacterial Activity and Cytotoxicity of Green Synthesized Composites Based on Silver Nanoparticles and Carboxymethyl-Cellulose

**DOI:** 10.3390/nano10061164

**Published:** 2020-06-14

**Authors:** María de los Ángeles Martínez-Rodríguez, Elizabeth Madla-Cruz, Victor H. Urrutia-Baca, Myriam A. de la Garza-Ramos, Virgilio A. González-González, Marco A. Garza-Navarro

**Affiliations:** 1 Facultad de Ingeniería Mecánica y Eléctrica, Universidad Autónoma de Nuevo León, San Nicolás de los Garza 66455, Nuevo León, Mexico; angelesmttz@live.com.mx (M.A.M.-R.); virgilio.gonzalezgnz@uanl.edu.mx (V.A.G.-G.); 2 Facultad de Ciencias Biológicas, Laboratorio de Inmunología y Virología, Universidad Autónoma de Nuevo León, San Nicolás de los Garza 66455, Nuevo León, Mexico; elizabethmadla@hotmail.com; 3 CHRISTUS—LATAM HUB Center of Excellence and Innovation, S.C. (CHRISTUS CEI), Monterrey 66260, Nuevo León, Mexico; vurrutia1990@gmail.com; 4 Centro de Investigación y Desarrollo en Ciencias de la Salud, Universidad Autónoma de Nuevo León, Unidad de Odontología Integral y Especialidades, Monterrey 64460, Nuevo León, Mexico; myriam.garzarm@uanl.edu.mx; 5 Centro de Innovación, Investigación y Desarrollo en Ingeniería y Tecnología, Universidad Autónoma de Nuevo León, Apodaca 66600, Nuevo León, Mexico

**Keywords:** silver nanoparticles, carboxymethyl-cellulose, composite, antibacterial activity, cytotoxicity

## Abstract

In this paper we report on the influence of polysaccharides’ molecular structure on the antibacterial activity and cytotoxicity of composites based on silver nanoparticles (AgNPs) immobilized into carboxymethyl-cellulose (CMC). These composites were green synthesized from the reduction of silver ions into aqueous solutions of the polysaccharide, using CMC with different degree of substitution (DS) and molecular weight (Mw). The composites were characterized by transmission electron microscopy (TEM), as well as infrared (ATR-FTIR), ultraviolet (UV-Vis), Raman, and X-ray photo-electron (XPS) spectroscopic techniques. The antibacterial activity was evaluated with minimum inhibitory concentration against *Enterococcus faecalis*. The cytotoxicity of composites was assessed against human gingival fibroblast. Experimental evidence suggests that particle size distribution and morphology of AgNPs change according to the quantity of silver precursor added to the reaction, as well as the DS and Mw of CMC used for composites preparation. This is related to the dispersion of silver precursor into aqueous solutions of the polysaccharide and the formation of Ag-O coordination bonds among AgNPs and COO^−^ moieties of CMC. Moreover, these coordination bonds modify the ability of nanoparticles to produce and release Ag^+^ into aqueous dispersion, adjusting their antibacterial activity and the induction of cytotoxicity into the tested biological environments.

## 1. Introduction

In recent years, nanotechnology has impacted the development of new functional materials based on nanostructures. Among the so-called nanomaterials, silver nanoparticles (AgNPs) have emerged as a promising specie to be used in biomedical and food packaging applications as a bactericide, fungicide, and antiviral [1,2,3,4]. There can be found studies regarding the effect of AgNPs on gram-negative bacteria, which indicate that nanoparticles with size between one and ten nanometers adhere to the surface of the bacteria cell membrane and disturb its permeability and respiration [5]. In addition, AgNPs dispersed into aqueous media can release Ag^+^ ions that can be internalized by passive bacterial transport through the channels in the cell membrane of both Gram-negative and positive bacteria [6]. The Ag^+^ ions inflict further damage to the bacteria due to its interaction with sulfur- and phosphorous- groups at the DNA, causing a loss in its ability to replicate; along with the deactivation of bacteria proteins, because of the interaction of Ag^+^ ions with their thiol (R-SH) groups [7].

Nonetheless, the main concern about the use of AgNPs in biomedical and food packaging applications is their toxicity. It has been reported that AgNPs display a size-dependent cytotoxicity, related to the generation of reactive oxygen species (ROS) during their surface oxidation and subsequent release of Ag^+^ ions into biological environments [8,9]. So, it is necessary to search for low-toxic AgNPs from methodologies that do not use nor produce toxic species. Accordingly, the “green” chemistry implies the design, development, and application of chemical products and process to reduce or eliminate the use or generation of hazardous substances to human health and the environment [10]. As has been reported in literature, the green chemistry routes for synthesis of AgNPs consider biopolymers such as chitosan, poly(lactic acid), sodium alginate, cellulose, and carboxymethyl-cellulose as both reducing and capping agents [11,12,13,14,15,16].

Among these biopolymers, the carboxymethyl-cellulose (CMC) emerges as a promising reducing and immobilization media for the green synthesis of AgNPs, due to its good chemical stability, as well as its biocompatible and biodegradable characteristics. The CMC is a semi-synthetic polysaccharide derived from the natural polymer cellulose, which undergoes the partial substitution of cellulose native hydroxymethyl (RCH_2_OH) groups by carboxymethyl (RCOOH) groups [17]. The degree of substitution (DS) of RCH_2_OH by RCOOH is reported as an average of carboxymethyl groups per monomer unit. The CMC is usually commercialized as a water-soluble sodium salt, which in aqueous solution can be loaded with metallic ions as Ag^+^ by a simple displacement reaction of Na^+^ [18]. Moreover, due to the abundant hydroxyl groups on its molecular structure, CMC has been successfully used as a reducing agent for the preparation of CMC-AgNPs composites [16,19]. From this approach is possible to get an outstanding particle size control and good efficiency over the silver ions reduction, without the use or generation of hazardous substances.

We previously reported on the ability of green synthesized CMC-AgNPs composites to inhibit the proliferation of Gram-positive and negative bacteria, such as *Streptococcus mutans* and *Porphyromonas gingivalis*, respectively, with a suitable cytotoxicity [20]. Nonetheless, currently, experimental evidence regarding the role of molecular structure of polysaccharides as CMC on the antibacterial activity and cytotoxicity of AgNPs-based composites does not exists. Consequently, in this work we report on the influence of polysaccharides’ molecular structure on the antibacterial activity and cytotoxicity of CMC-AgNPs composites synthesized from a green chemistry route, by the use of CMC with different DS and molecular weight (Mw) as a reducing agent and immobilization media.

## 2. Materials and Methods

### 2.1. Synthesis and Characterization of CMC-AgNPs Composite

The CMC with DS = 0.7 and Mw = 90 kDa (0.7CMC), CMC with DS = 0.9 and Mw = 250 kDa (0.9CMC), CMC with DS = 1.2 and Mw = 250 kDa (1.2CMC) and silver nitrate (AgNO_3_) were purchased from Sigma-Aldrich Co., Edo. de México, México, and used as received without any further treatment for the synthesis of CMC-AgNPs composites. Deionized water was used for the preparation of all solutions for this investigation (Barnstead EASYpure II system with ρ = 13 MΩ-cm).

The synthesis of CMC-AgNPs composites was performed following a previously reported route, with some modifications [19]. Briefly, aqueous CMC and AgNO_3_ solutions were prepared at concentrations of 15 mg/mL and 0.24, 0.48, 0.94, or 1.26 mg/mL, respectively, using deionized water. Then, 20 mL of CMC was added into a round-bottom three-neck flask (reactor) and stirred for 10 min under room conditions. Later, 10 mL of AgNO_3_ solution was added to the reactor and the temperature was raised to 90 °C. The reaction was kept at this temperature for 24 h under reflux conditions. After 24 h, the resultant yellowish to reddish dispersions (depending on the concentration of AgNO_3_ solution added to the reaction) was poured into a previously cooled round-bottom flask, in order to rapidly lower its temperature towards room temperature. These dispersions were frozen and then lyophilized. This process was performed using aqueous solutions of 0.7CMC, 0.9CMC, or 1.2CMC at a constant concentration of 15 mg/mL; as well as AgNO_3_ solutions at the aforementioned concentrations of 0.24, 0.48, 0.94, or 1.26 mg/mL to obtain the composite samples 0.7/0.9/1.2Ag1, 0.7/0.9/1.2Ag2, 0.7/0.9/1.2Ag3, or 0.7/0.9/1.2Ag4, respectively. The Table 1 shows the CMC and AgNO_3_ weights that were added to the reactor for the synthesis of each sample. Finally, dried samples were weighted and dissolved in deionized water to prepare CMC-AgNPs composites’ dispersions for their further characterization. 

The crystalline and morphological features of CMC-AgNPs composites were examined by transmission electron microscopy (TEM) in a Field Emission Gun, FEI Titan G2 80-300 microscope, using electron microscopy techniques as bright field (BF) and Z-contrast (HAADF-STEM) imaging, as well as selected area electron diffraction (SAED). Particle size distribution of AgNPs was obtained from the measuring of at least 300 randomly selected particles in CMC-AgNPs samples using Graphic for Mac 3.1 software; and adjusting the experimental measuring data to the Gaussian statistic model in OriginPro 8.5.0 software, using tools as a descriptive statistic (frequency counts) and analysis (fitting). Ultraviolet-visible spectroscopy (UV-vis) studies of CMC-AgNPs composites as well as AgNO_3_ precursor solution were performed in a Perkin-Elmer, Lambda 35, spectrometer to evaluate the reduction efficiency of the proposed synthesis route. Interactions between CMC molecules and AgNPs were examined using infrared spectroscopy (ATR-FTIR). ATR-FTIR spectra of pure 0.7CMC, 0.9CMC, 1.2CMC, as well as CMC-AgNPs samples were recorded in a Frontier MIR FT-IR, Universal ATR spectrometer. In addition, Raman spectroscopy was carried out in a Thermo Scientific, DXR Raman microscope. The spectra of selected composite samples were measured after 30 s of exposure and acquisition time of 60 s, using a radiation of 532 nm. Finally, X-Ray Photoelectron Spectroscopy (XPS) was perform for the measuring of C1s, O1s, and Ag3d spectra for pure 0.7CMC, 0.9CMC, and 1.2CMC, as well as for selected CMC-AgNPs samples in a Thermo-Scientific, K-Alpha spectrometer with monochromatized AlKα radiation (E = 1.5 keV), X-ray spot of 400 μm, and flood gun for charge compensation.

### 2.2. Antibacterial Assay

The antibacterial activity of CMC-AgNPs composites was examined using the standard broth dilution method. The minimal inhibitory concentration (MIC) was determined from 96-well flat-bottom plates containing 50 μL of CMC-AgNPs dilutions with concentrations [AgNPs] = 60 to 3.75 µg/mL in Brain Heart Infusion (BHI) medium (Becton Dickinson Bioxon, Edo. de México, México); and 50 μL of 1.0 × 10^8^ CFU/mL of *Enterococcus faecalis* (ATCC^®^ 29212™) (*E. faecalis*), up to a final volume of 100 μL per well. In addition, ampicillin at 5 μg/mL was used as a positive control for inhibition of bacterial growth; whereas BHI medium was employed as negative control. The prepared cultures were incubated at 37 °C for 24 h in an aerobic atmosphere. Bacterial growth was measured from the absorbance of the cultures at 595 nm using an iMark™ microplate reader (Bio-Rad laboratories, Hercules, CA, USA). Subsequently, the percentage of growth inhibition was calculated using:

(1) $$\%\;inhibition=100-\left\{\left[\frac{\left(Sample-Positive\;control\right)}{\left(Negative\;control-Positive\;control\right)}\right]\times 100\right\}$$

The MIC value was defined as the lowest concentration of CMC-AgNPs that inhibited 99% of growth bacterial.

### 2.3. Cytotoxicity Assay

The cytotoxicity of CMC-AgNPs composites was evaluated against human gingival fibroblast cells (ATCC^®^PCS-201-018™), from 3-(4, 5-dimethylthiazol-2-yl)-2, 5-diphenyl tetrazolium (MTT) assay. The cell line was cultured in Dulbecco’s modified Eagle’s medium (DMEM), supplemented with 10% FBS, 1X antibiotic-antimycotic, and 6 mM L-glutamine (complete-DMEM) at 37 °C for 48 h in a humidified atmosphere of 5% CO_2_. Later, 100 μL of complete-DMEM containing 5 × 10^4^ cells were placed into each well of a flat-bottom 96-well plate and grown to approximately 90% confluence. Then, 100 µL of CMC-AgNPs dilutions at [AgNPs] = 60 to 3.75 µg/mL were added to each well and incubated for 24 h. Complete-DMEM and 2% Triton X-100 were used as negative and positive control, respectively. After incubation, supernatant was discarded, and the cells were carefully washed with PBS. Later, 100 μL of MTT diluted in complete-DMEM at 0.5 mg/mL were added to the wells and the cultures were incubated for 4 h. Subsequently, the supernatant was discarded, and the resulting formazan crystals were solubilized with 200 μL dimethyl sulfoxide. Finally, the absorbance of the cultures was recorded at 570 nm using a microplate reader. The percentage of cytotoxicity was calculated using:

(2) $$\%\;cytotoxicity=100-\left\{\left[\frac{\left(Sample-Positive\;control\right)}{\left(Negative\;control-Positive\;control\right)}\right]\times 100\right\}$$

## 3. Results and Discussion

### 3.1. Morphological and Crystalline Features of CMC-AgNPs Composites

**Figure 1:**
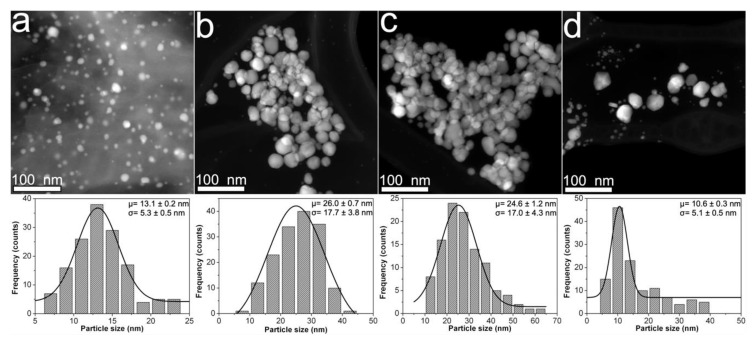
HAADF-STEM images obtained from composites samples: (**a**) 0.7Ag1; (**b**) 0.7Ag2; (**c**) 0.7Ag3; and (**d**) 0.7Ag4. The particle size distribution of each sample is shown just below its HAADF-STEM image.

Figure 1 shows HAADF-STEM images taken from CMC-AgNPs composites that were synthesized using 0.7CMC as a reducing and immobilization agent. As Figure 1a displays, nanoparticles in sample 0.7Ag1 show a quasi-spherical morphology. There is also presented the adjustment of experimental data from particle size measuring to the Gaussian statistic model. The center of particle size distribution is 13.1 nm, showing a standard deviation of 5.3 nm. Nonetheless, the statistical distributions obtained for samples 0.7Ag2 and 0.7Ag3 depict centers at a larger particle size of 26 and 24.6 nm, respectively; as well as wider dispersions, with standard deviations of 17.7 and 17.0 nm, respectively (see Figure 1b,c). In addition, the nanoparticles in samples 0.7Ag2 and 0.7Ag3 show a change on their morphology from quasi-spherical to one that displays facets. The presence of faceted nanoparticles is also observed in sample 0.7Ag4, along with a large population of small nanoparticles with a mean size of 10.6 nm and standard deviation of 5.1 nm (see Figure 1d). The change in particle size distribution and morphology of nanoparticles could be related to a decrease in the ability of CMC to control their growth as the weight content of AgNO_3_ is increased (see Table 1). 

**Figure 2:**
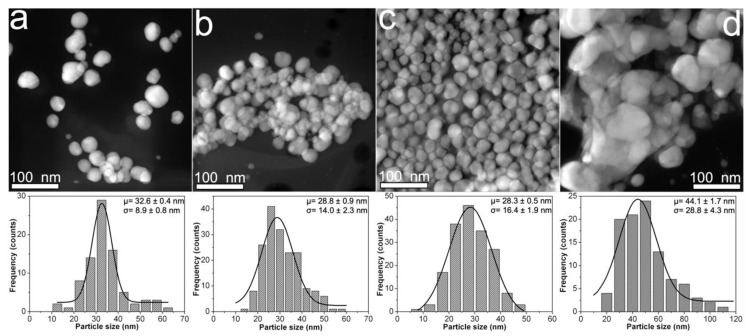
HAADF-STEM images obtained from composites samples: (**a**) 0.9Ag1; (**b**) 0.9Ag2; (**c**) 0.9Ag3; and (**d**) 0.9Ag4. The particle size distribution of each sample is shown just below its HAADF-STEM image.

The Figure 2 displays the morphological features of CMC-AgNPs samples that were obtained from 0.9CMC aqueous solutions. Herein we observed that mean particle size shows a small decrease from 32.6 to 28.3 nm as the weight content of AgNO_3_ used for the synthesis of 0.9Ag1, 0.9Ag2, and 0.9Ag3 increases (see Table 1); as well as an increase in the standard deviation from 8.9 to 16.4 nm (see Figure 2a–c). Moreover, the nanoparticles in sample 0.9Ag4 display a remarkable increase in both mean particle size (44.1 nm) and standard deviation (28.8 nm) (see Figure 2d). The formation of aggregates from faceted nanoparticles in 0.9Ag4 is also noticeable. According to these results, the 0.7CMC reagent gives smaller mean particle size but larger standard deviation than 0.9CMC reagent when 2.41, 4.82, or 9.45 mg of AgNO_3_ are used for the synthesis of samples.

**Figure 3:**
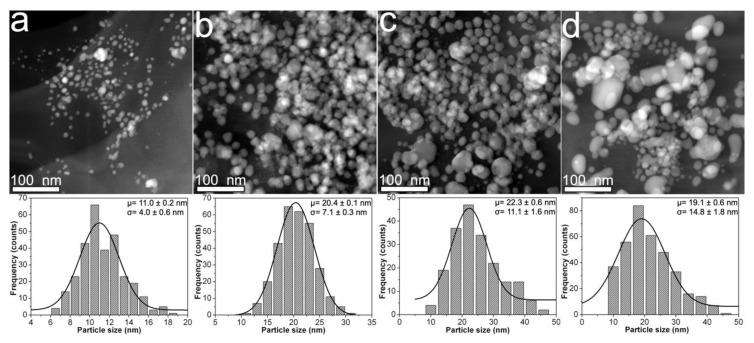
HAADF-STEM images obtained from composites samples: (**a**) 1.2Ag1; (**b**) 1.2Ag2; (**c**) 1.2Ag3; and (**d**) 1.2Ag4. The particle size distribution of each sample is shown just below its HAADF-STEM image.

Figure 3 shows the morphological characteristics for samples prepared with 1.2CMC reagent. For this case, the mean particle size tends to increase from 11 to 22.3 nm for samples 1.2Ag1, 1.2Ag2, and 1.2Ag3 (see Figure 3a–c) as the weight content of AgNO_3_ increases (see Table 1). Nonetheless, this trend is not followed by 1.2Ag4, since it displays a mean particle size of 19.1 nm (see Figure 3d). The presence of nanoparticle aggregates that resemble those observed in the 0.9Ag4 sample is also seen in Figure 3d. Finally, the standard deviation for this experimental set varies in a direct proportion with the weight of AgNO_3_ added for the synthesis of samples 1.2Ag1, 1.2Ag2, 1.2Ag3, and 1.2Ag4; and covers an interval from 4 to 14.8 nm. This evidence suggests that 1.2CMC reagent provides better control on particle size distribution than 0.7CMC and 0.9CMC reagents at the CMC/AgNO_3_ weight ratios used for sample preparation (see Table 1). Table 2 reports the data from particle size distribution that were obtained for the synthesized composite samples.

**Figure 4:**
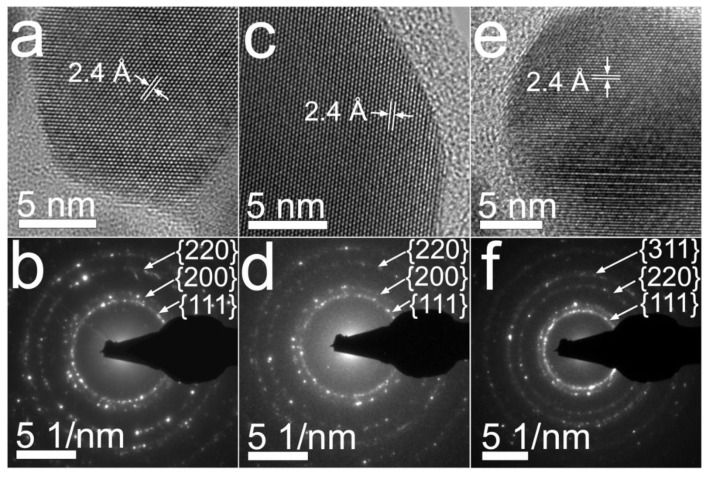
BF images and SAED patterns obtained from samples: (**a**) and (**b**) 0.7Ag1; (**c**) and (**d**) 0.9Ag1; (**e**) and (**f**) 1.2Ag1.

Figure 4 resumes the crystalline features that were observed for nanoparticles prepared from aqueous solutions of 0.7CMC, 0.9CMC, and 1.2CMC. As Figure 4a,c,e display, the nanoparticles from samples 0.7Ag1, 0.9Ag1, and 1.2Ag1, respectively, depict a regular atomic arrangement, showing lattice fringes with a regular interplanar spacing of 2.4 Å. This spacing is congruent with that reported for family planes {111} of the crystalline structure of silver (see JCPDS: 04-0783). Furthermore, in the SAED patterns reported in Figure 4b,d,f we recognize diffraction rings related to family planes {111}, {200}, {220}, and {311} of the face-centered cubic (FCC) packing of silver (see JCPDS: 04-0783). This evidence confirms the formation of AgNPs in the synthesized samples.

**Figure 5:**
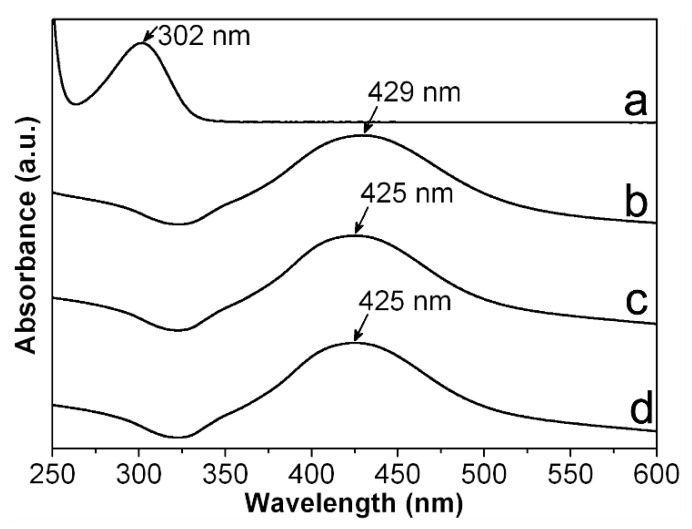
UV-vis spectra measured from (**a**) AgNO_3_; (**b**) 0.7Ag4; (**c**) 0.9Ag4; and (**d**) 1.2Ag4.

However, in order to obtain a first approach regarding the reduction efficiency of Ag^+^ from our synthesis route, we record UV-vis spectra for CMC-AgNPs composites and AgNO_3_ solution used for their synthesis. As it can be observed in Figure 5, the spectra obtained from CMC-AgNPs composites do not show the absorption band related to the Ag^+^ at 301 nm (see Figure 5a); instead, they display a well-defined band around 425–429 nm (see Figure 5b–d). According to the literature, this band is related to the characteristic surface plasmon resonance of AgNPs [21]. This result indicates that there are no detectable traces related to Ag^+^ ions in the analyzed samples, suggesting full reduction of added Ag^+^ into Ag^0^. Accordingly, the CMC/AgNPs weight ratio could be calculated as 200, 100, 50, and 37.5 for the composites 0.7/0.9/1.2Ag1, 0.7/0.9/1.2Ag2, 0.7/0.9/1.2Ag3, and 0.7/0.9/1.2Ag4, respectively (see Table 1). Nonetheless, to get more information about the immobilization features of the different kind of CMC, it is necessary to evaluate the manner that the polysaccharide’s chains interacts with the synthesized AgNPs. Consequently, we proceed to perform ATR-FTIR measures of CMC reagents and CMC-AgNPs samples. 

### 3.2. Spectroscopic Characterization

**Figure 6:**
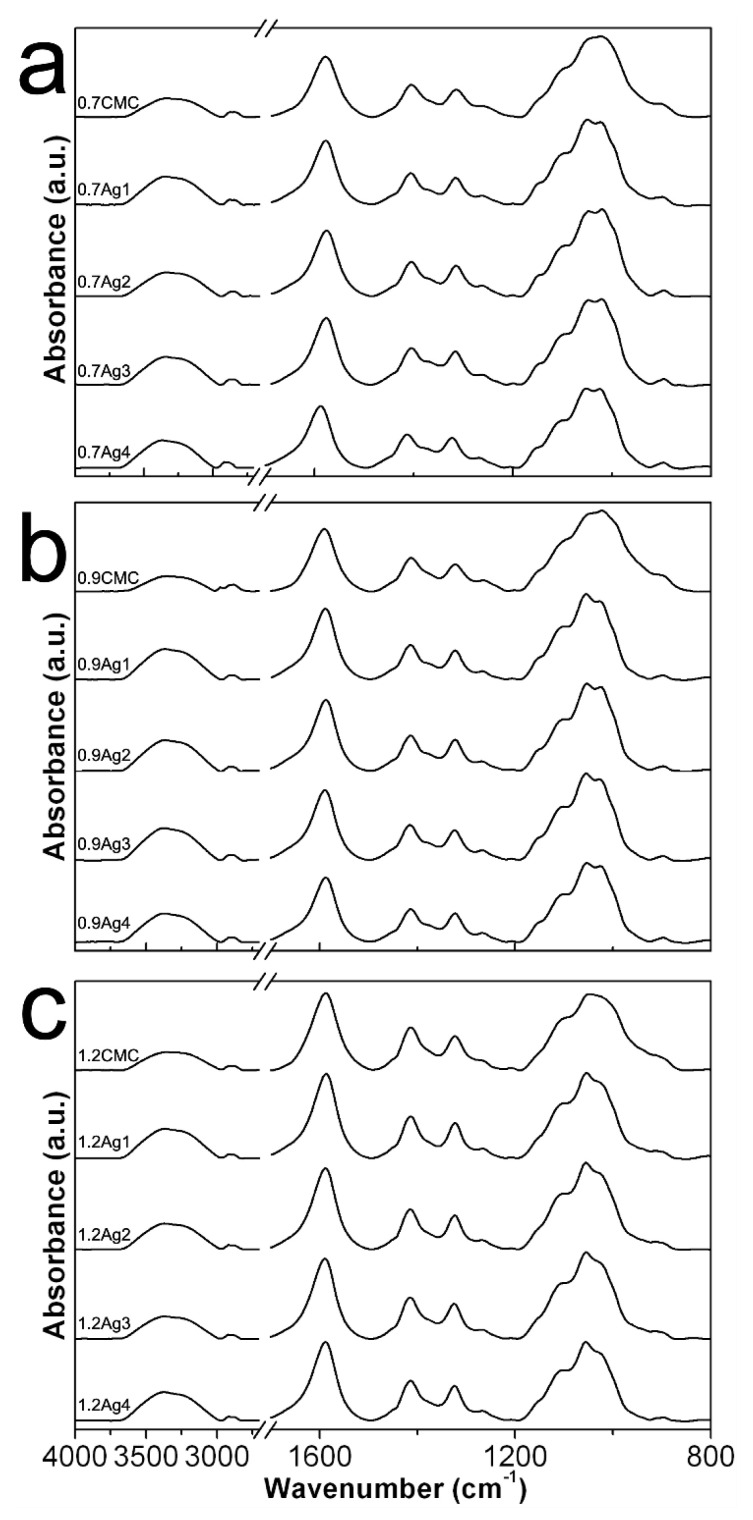
ATR-FTIR spectra obtained from (**a**) pure 0.7CMC and its composite samples; (**b**) pure 0.9CMC and its composite samples; (**c**) pure 1.2CMC and its composite samples.

Figure 6 shows the ATR-FTIR spectra obtained from powdered 0.7CMC, 0.9CMC, and 1.2CMC reagents, as well as those recorded from powdered CMC-AgNPs samples. Figure 6a displays the spectrum obtained for 0.7CMC, where it can recognize absorption bands related to [18,22,23]: symmetrical and asymmetrical stretching at O-H bond of hydroxyl groups (R-OH) at 3360 cm^−1^; asymmetrical stretching at the C-H bond of the hydroxymethyl functional groups (R-CH_2_OH) at 2911 cm^−1^; asymmetrical and symmetrical stretching of -O-C = O bonds on the carboxymethyl functional groups (R-CH_2_OCOO^−^) at 1590 cm^−1^ and 1413 cm^−1^, respectively; bending of -C-CH and O-CH- bonds on the R-CH_2_OCOO^−^ groups at 1321 cm^−1^; stretching of C-O bond on R-CH_2_OCOO^−^ at 1269 and 1026 cm^−1^; and stretching of C-O-C bonds on R-CH_2_OCOO^−^ at 1099 and 1043 cm^−1^. Figure 6a also shows the spectra recorded from samples 0.7Ag1, 0.7Ag2, 0.7Ag3, and 0.7Ag4. Herein we noticed a slight bathochromic shift in the position of the band related to asymmetrical stretching of O-C = O moieties at R-CH_2_OCOO^−^, from 1590 to 1586 cm^−1^; along with a hypsochromic one from 1043 to 1053 cm^−1^ of the band associated to stretching on C-O-C bonds at the same functional groups.

In addition, Figure 6b depicts the spectrum obtained from powdered 0.9CMC, as well as those from composites 0.9Ag1, 0.9Ag2, 0.9Ag3, and 0.9Ag4. Likewise, bands related to vibrational modes of 0.9CMC molecules can be noticed, as the asymmetrical stretching of -O-C = O moieties at 1590 cm^−1^ and stretching of C-O-C bonds at 1044 cm^−1^, which display bathochromic and hypsochromic shifts towards 1587 cm^−1^ and 1050 cm^−1^, respectively, in the composites spectra. Moreover, this phenomenon also occurs for the samples 1.2Ag1, 1.2Ag2, 1.2Ag3, and 1.2 Ag4, since they display a noticeable shift on the band related to the stretching of C-O-C, from 1049 to 1055 cm^−1^, with respect the position of this band in the spectrum recorded for 1.2CMC (see Figure 6c). These features suggest an interaction between CMC molecules and AgNPs for all the samples, which could be attributed to the adsorption of R-CH_2_OCOO^−^ onto nanoparticles, as it has been reported elsewhere [24,25,26]. 

**Figure 7:**
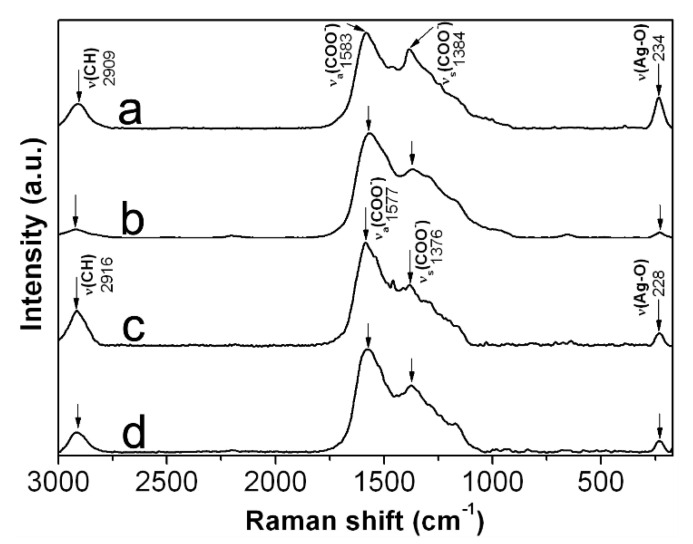
Raman spectra recorded from: (**a**) 0.7Ag1; (**b**) 0.7Ag4; (**c**) 1.2Ag1; and (**d**) 1.2Ag2.

In order to corroborate the adsorption of CMC chains onto AgNPs, we record Raman spectra from selected powdered samples. Figure 7 shows the Raman spectra obtained from 0.7Ag1, 0.7 Ag4, 1.2Ag1, and 1.2Ag2. Here it is possible to identify bands associated with vibrational modes of CMC, such as stretching of C-H at 2916–2909 cm^−1^; as well as asymmetrical and symmetrical stretching of O-C = O at 1588–1577 cm^−1^ and 1384–1376 cm^−1^, respectively [25,26,27,28]. An increase in the intensity of Raman scattering in the bands attributed to stretching vibration in O-C = O can also be noticed, which seems to be related with the increase in the weight content of AgNPs in samples. The increase in intensity of both bands was evaluated taking as reference the intensity of the band attributed to C-H stretching vibration. As it has been documented in literature, the increase in the intensity of these bands can be related to the adsorption of COO^−^ moieties onto metal or semimetal nanoparticles; and occurs due to electric field induced surface enhanced Raman scattering (SERS) [29]. Moreover, there can be found a band at 234–228 cm^−1^, attributed to the stretching vibration of Ag-O bond [30,31]. These results confirm the adsorption of CMC chains onto AgNPs and suggest the formation of a bond between Ag and O in the COO^−^ moieties of CMC, as it has been proposed elsewhere [30].

**Figure 8:**
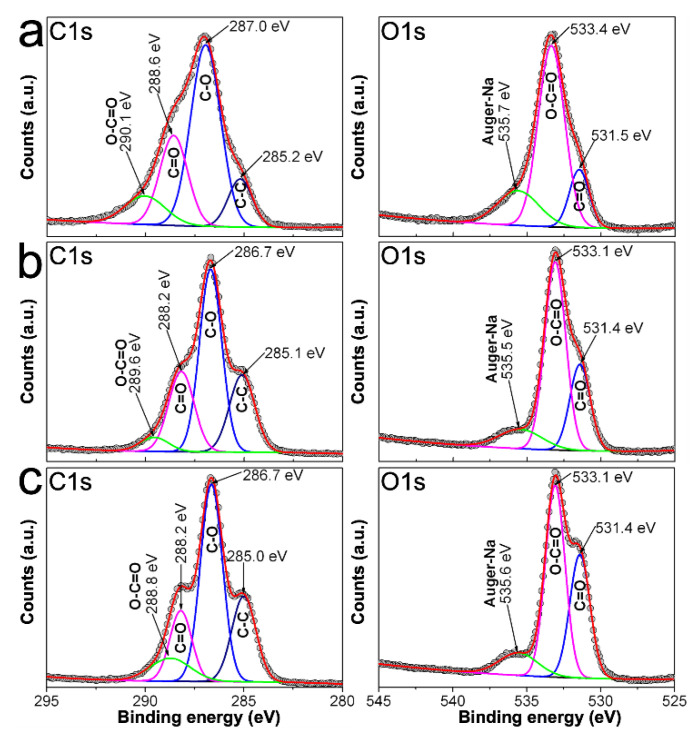
C1s and O1s spectra recorded by XPS from: (**a**) 0.7CMC; (**b**) 0.9CMC; and (**c**) 1.2CMC.

In order to get further insight regarding the adsorption of RCH_2_OCOO^−^ onto AgNPs surface, we proceed to measure C1s, O1s, and Ag3d XPS spectra from 0.7CMC, 0.9CMC, 1.2CMC reagents, as well as from some powdered samples. Peaks of the recorded XPS spectra were deconvoluted and fitted using a Gaussian approach in PeakFitV4.12 software. Accordingly, Figure 8a shows C1s and O1s spectra recorded from 0.7CMC. The C1s spectrum exhibits four peaks at 285.2, 287.0, 288.6, and 290.1 eV, which can be attributed to C in C-C, C-O, C = O, and O-C = O, respectively. Three peaks are observed in O1s spectrum that can be attributed to C = O, O-C = O and Auger electrons from Na at 531.5, 533.4, and 535.7 eV, respectively. Likewise, C1s spectra recorded from 0.9CMC and 1.2CMC were deconvoluted into four peaks at 285.0 eV (C-C), 286.7 eV (C-O), 288.2 eV (C = O), and 289.6 or 288.8 eV (O-C = O); whereas their O1s spectra show peaks at 531.4 eV (C = O), 533.1 eV (O-C = O), and 535.6 eV (Auger-Na) (see Figure 8b,c). The presence of these signals agrees with those expected from the molecular structure of these polysaccharides [32,33].

**Figure 9:**
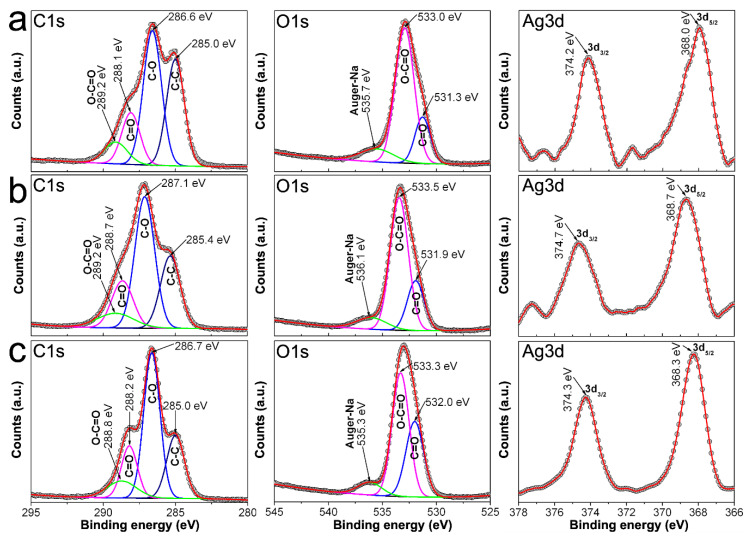
C1s and O1s spectra recorded by XPS from: (**a**) 0.7Ag1; (**b**) 0.9Ag1; and (**c**) 1.2Ag1.

Figure 9 show C1s, O1s, and Ag3d spectra from samples 0.7Ag1, 0.9Ag1, and 1.2Ag1. C1s spectra of samples show peaks related to C-C, C-O, C = O, and O-C = O of the polysaccharide’s chains, although they display changes in their binding energies with respect to that obtained from CMC reagents (see Figure 8); along with a change in the intensity of each peak (quantity of photoelectrons emitted from samples). Changes in binding energy occur into an interval from 0.2 to 0.9 eV, and are more obvious for emissions associated with C = O and O-C = O. In addition, O1s spectra show shifts in the binding energies related to emissions from C = O and O-C = O, into an interval between 0.4 and 0.9 eV. There is also observed Ag3d spectra of these samples that display peaks at 374.2–374.7 eV and 368.0–368.7 eV, which correspond to photoelectrons emitted from 3d_3/2_ and 3d_5/2_ states, respectively. The difference between the binding energies of such emissions is 6 eV for all cases, confirming that silver in samples is only Ag^0^ [34,35,36]. Moreover, this experimental evidence is congruent with that obtained from UV-vis spectra regarding the full reduction of Ag^+^ into Ag^0^ (see Figure 5).

**Figure 10:**
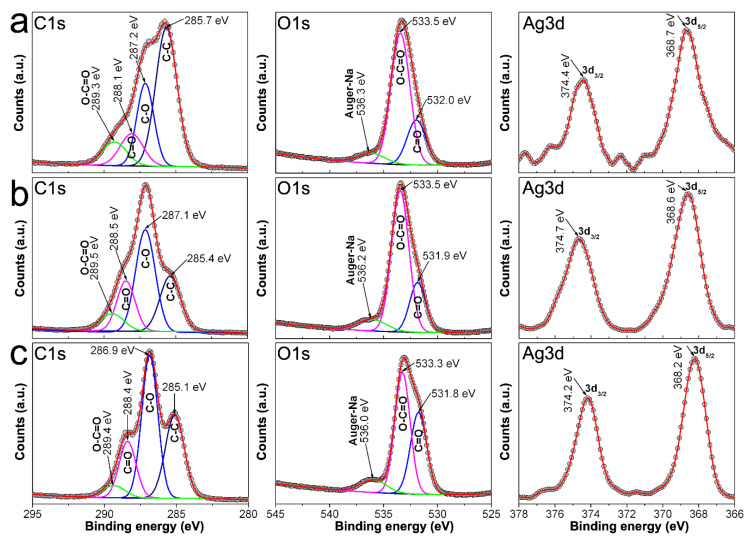
C1s, O1s and Ag3d spectra recorded by XPS from samples: (**a**) 0.7Ag2; (**b**) 0.9Ag2; and (**c**) 1.2Ag2.

Likewise, Figure 10 shows the XPS spectra recorded from samples 0.7Ag2, 0.9Ag2, and 1.2Ag2. Herein, C1s and O1s spectra depict shifts in the binding energies related to C and O in C = O and O-C = O bonds that reach up 0.9 eV, with respect to those observed in Figure 8; as well as peaks around 374 and 368 eV in their Ag3d spectra, related to core emissions from 3d_3/2_ and 3d_5/2_, respectively. Thus, considering that the energy of photoelectrons emitted from discrete states as 1s is quite susceptible to change depending on the bonds that elements form, the fact that peaks related to C-C, C-O, O-C = O, and C = O display shifts on their binding energies indicates that AgNPs are immobilized in CMC by coordination bonds [36,37]. Moreover, taking into account the results obtained from Raman spectra, the coordination bonds can be attributed to those Ag-O among AgNPs and COO^−^ moieties of CMC.

Considering our experimental findings, the variation of the morphological features of AgNPs can be explained as follows. The CMC is capable of attracting Ag^+^ ions to intermolecular sites nearby the negatively charged R-CH_2_OCOO^−^ when both are diluted in aqueous media [18]. In these sites, the Ag^+^ ions are reduced with the electrons realized from R–OH or R–CH_2_OH groups of CMC at high temperature (i.e., 90 °C). Accordingly, the coalescence of Ag^0^ conduces to the nucleation and subsequent growth of AgNPs, which will depend on the quantity of silver reagent added to the solution [19]. Hence, it is possible to state that when a CMC with a given DS and Mw is used for the synthesis of AgNPs, their particle size could increase as the weight content of AgNO_3_ added to the reaction increases. This could explain the particle size distributions (mean size and standard deviation) obtained from almost the samples prepared from 0.9CMC and 1.2CMC aqueous solutions (see Figure 2 and Figure 3). In addition, we observe that samples prepared with the same weight content of AgNO_3_ but different CMC reagent display variations in their particle size distribution and morphology. According to our experimental evidence, the AgNPs are susceptible to form coordination bonds with COO^−^ moieties. Therefore, the particle size distribution and morphology of AgNPs will depend on the quantity of R-CH_2_OCOO^−^ available for their immobilization. The quantity of R-CH_2_OCOO^−^ available to restrict the size of AgNPs and avoid their secondary growth varies according to the DS of 0.9CMC and 1.2CMC (see Table 2). 

This explanation seems to disagree with the morphological features of samples obtained from 0.7CMC aqueous solutions, since their particle size distributions (mean size and standard deviation) do not change in direct proportion with the weight of AgNO_3_ added to each reaction; and they display smaller mean particle size than samples prepared with 0.9CMC reagent at the same weight content of AgNO_3_ (see Table 2). In order to explain this phenomenon, we should consider the following. As Table 1 shows, all CMC aqueous solutions were prepared at the same concentration for the synthesis of samples. However, the 0.7CMC reagent has a lower Mw than 0.9CMC and 1.2CMC. It is well known that viscosity of a polymer solution varies in a direct proportion with its Mw at a given concentration. So, it is reasonable to think that, at the same weight content of AgNO_3_, the dispersion of Ag^+^ ions in 0.7CMC aqueous medium differs from that in 0.9CMC or 1.2CMC solutions. Moreover, considering the low DS of 0.7CMC, it is possible that some Ag^+^ ions do not reach a site nearby the R-CH_2_OCOO^−^ groups; hence, they could be reduced elsewhere. This implies that the coalescence of Ag^0^, nucleation and subsequent growth of AgNPs also occur far from the R-CH_2_OCOO^−^ groups. This could explain the high standard deviation obtained from samples 0.7Ag2 and 0.7Ag3, as well as the formation of large and faceted nanoparticles in sample 0.7Ag4 (see Figure 1). Nonetheless, the AgNPs are immobilized in CMC by coordination bonds, thus, it is possible to argue that these Ag-O bonds among AgNPs and COO^−^ moieties restrict the growth for a large number of nanoparticles when 0.7CMC reagent is used for preparation of composite samples (see Figure 1). This could explain the small mean particle size obtained at low weight content of AgNO_3_ added for the preparation of 0.7Ag1 (see Table 2).

Therefore, it can be concluded that the key factor for the control of particle size distribution of synthesized AgNPs is the quantity of R-CH_2_OCOO^−^ available for their immobilization. The quantity of these groups varies in direct proportion with the DS of CMC, which in general gives smaller particle sizes for CMC with higher DS. It is worth mentioning that the observed trends regarding the changes on standard deviation and morphology of AgNPs, as well as their plausible explanations, should be confirmed in further studies.

### 3.3. Antibacterial Activity

**Figure 11:**
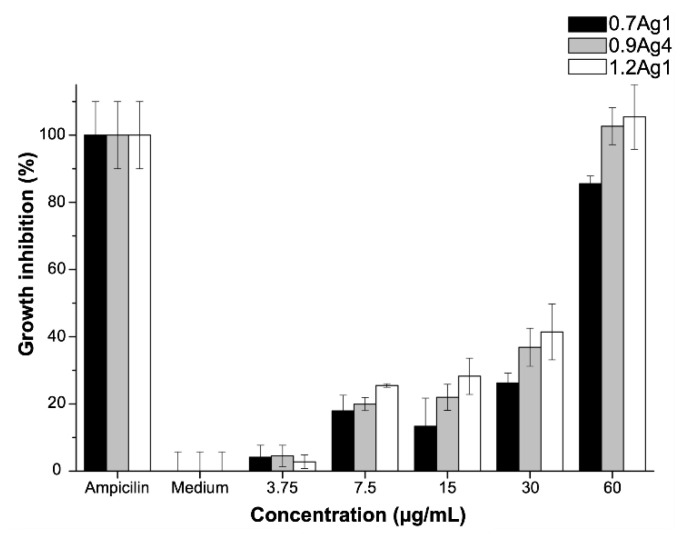
Antibacterial activity of CMC-AgNPs against *E. faecalis* growth. The data represent the percentage mean ± the percentage deviation.

As we explain in Section 3.2, particle size distribution and morphology of the AgNPs mainly vary according to the DS of CMC used for their synthesis. This is related to the fact that nanoparticles are immobilized in CMC by the formation of Ag-O coordination bonds among AgNPs and COO^−^ moieties of the polysaccharide’s chains. Thus, in order to address the effect of these bonds on the antibacterial activity of AgNPs, we proceed to test samples with similar particle size distributions but with nanoparticles immobilized into CMC with distinct DS, 0.7Ag1 and 1.2Ag1; along with the sample 0.9Ag4, which displays a different particle size distribution for nanoparticles immobilized into CMC with close DS to 1.2CMC. The antibacterial activity assays were performed by three replicates of three independent experiments using doses with a known concentration of AgNPs, [AgNPs]. As Figure 11 shows, an important antibacterial activity of CMC-AgNPs composites was observed at [AgNPs] = 60 µg/mL for the three tested samples. At this dose no statistical difference was observed compared to 5 μg/mL ampicillin (*p* > 0.05) for 0.9Ag4 and 1.2Ag1 samples. Therefore, [AgNPs] = 60 μg/mL can be established as the MIC value for our experimental setup, except for 0.7Ag1 sample. The sample 0.7Ag1 shows an inhibitory effect of 85.5 ± 2.3% at this dose. In addition, a residual inhibitory effect was observed at [AgNPs] = 30 µg/mL in 0.7Ag1, 0.9Ag4, and 1.2Ag1 with 26.3 ± 2.9%, 36.9 ± 5.7%, and 41.4 ± 8.3%, respectively (see Figure 11).

These results can be explained as follows. The antibacterial activity of AgNPs is believed to be related to the production and release of positive charged Ag ions from their surface in aqueous media [6]. Thus, smaller particle size leads to a large surface area, hence, to produce a higher amount of Ag^+^ in aqueous solution than larger AgNPs [38]. The Ag^+^ ions can be internalized by passive bacterial transport through the channels in the cell membrane bacteria and inflict damage due to their binding to cellular structural elements such as enzymes and proteins, particularly to their R-SH groups [6,7]. This binding diminishes the membrane permeability and leads to cell death [39]. Specifically, MIC value against gram-positive *E. faecalis* bacteria has been found in an interval between 500 and 0.19 μg/mL, depending on synthesis route, particle size, and surface modification [39,40,41,42]. Accordingly, the Ag-O coordination bonds among AgNPs and COO^−^ moieties of CMC do not blur the ability of nanoparticles to produce and release Ag^+^ from their surface, thus, to display a remarkable antibacterial activity in aqueous media. 

Moreover, it can be noticed that AgNPs with quite different particle size distribution but immobilized in CMC with close DS, as is the case for 0.9Ag4 and 1.2Ag1, display almost the same inhibition of bacteria growth (see Figure 11). This suggests that Ag-O coordination bonds among AgNPs and COO^−^ moieties of these polysaccharides enhance the antibacterial activity for CMC-AgNPs composites. This feature is congruent with the antibacterial activity observed for 0.7Ag1, since this sample presents a lower inhibitory effect than 1.2Ag1, even though both have a similar particle size distribution (see Table 2).

### 3.4. Cytotoxicity

**Figure 12:**
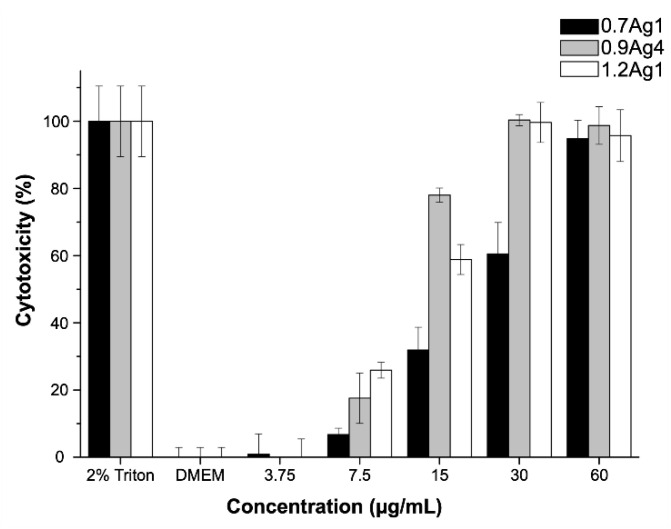
Cytotoxicity obtained from CMC-AgNPs composites against ATCC^®^PCS-201-018™ cell line. The data represent the percentage mean ± the percentage deviation.

Considering the inhibitory effect on the bacteria growth of samples 0.7Ag1, 0.9Ag4, and 1.2Ag1, we proceed to evaluate their cytotoxicity at the same tested doses for the antibacterial assays. The cytotoxicity assays were performed by three replicates of three independent experiments for the tested samples. As can be noticed in Figure 12, the highest cytotoxic effect occurs at [AgNPs] = 60 µg/mL for the three samples of CMC-AgNPs, showing a cytotoxicity greater than 95% after 24 h of treatment. However, the cytotoxic effect decreased in a dose dependent manner. At 30 µg/mL, 0.7Ag1 shows a lower cytotoxicity (60.5 ± 9.4%) than that from 0.9Ag4 and 1.2Ag1 of 100.3 ± 1.7% and 99.7 ± 5.9%, respectively. For subsequent dilutions, the decrease in cytotoxicity was more pronounced for 0.7Ag1 compared to the other two samples (*p* < 0.001). In addition, no cytotoxic effect was observed at a dilution of [AgNPs] = 3.75 μg/mL for the tested samples, since there are not statistical differences compared to the complete-DMEM control (*p* > 0.05). It is worth mentioning that at this dose no significant antibacterial activity was observed (see Figure 11).

In order to explain these results, we should consider the following. It is well known that AgNPs-mediated cytotoxicity in mammalian cells depends greatly on the nanoparticle size, shape, surface charge, dosage, oxidation state, and agglomeration condition as well as the cell type. Moreover, it has been demonstrated that antibacterial activity of AgNPs in aqueous media is related to oxidation of their surface and subsequent release of Ag^+^ [43]. This oxidation conduces to the formation of reactive oxygen species (ROS) which trigger several negative effects on cell structures and their functions, inducing cytotoxicity [6,44]. Accordingly, the fact that 0.7Ag1 displays lower toxicity than 0.9Ag4 and 1.2Ag1 at all tested doses suggests that generation of ROS is diminished by the use of CMC with low DS for AgNPs immobilization. This is congruent with the results obtained from antibacterial activity assays regarding the inhibition of bacteria growth.

Hence, it can be concluded that Ag-O coordination bonds among AgNPs and COO^−^ moieties of CMC modify the ability of nanoparticles to produce and release Ag^+^ into aqueous dispersion, adjusting their antibacterial activity and the induction of cytotoxicity into the tested biological environments. Finally, Table 3 summarize the results obtained from this work.

## 4. Conclusions

The influence of polysaccharides’ molecular structure on the antibacterial activity and cytotoxicity of green synthesized composites based on AgNPs immobilized into CMC was reported. The experimental evidence suggests that the particle size distribution and morphology of AgNPs mainly depend on the quantity of R-CH_2_OCOO^−^ groups available for their immobilization. This is related to the fact that nanoparticles are immobilized in CMC by the formation of Ag-O coordination bonds among AgNPs and COO^−^ moieties of the polysaccharide’s chains. Accordingly, the quantity of R-CH_2_OCOO^−^ groups varies in direct proportion with the DS of CMC, which in general gives smaller particle size for CMC with higher DS. Moreover, the biological assays indicate that the antibacterial activity and cytotoxicity of the tested samples increase by the use of CMC with higher DS as AgNPs immobilization medium. Hence, it can be concluded that Ag-O coordination bonds among AgNPs and COO^−^ moieties of CMC modify the ability of nanoparticles to produce and release Ag^+^ into aqueous dispersion, adjusting their antibacterial activity and the induction of cytotoxicity into the tested biological environments. Finally, it is worth mentioning that the width of the particle size distribution and morphology of AgNPs also depends on the weight of AgNO_3_ added to the reaction and the Mw of CMC used for their synthesis. This could be related to the manner that silver ions are dispersed into the used CMC aqueous solutions for their reduction and subsequent nucleation and growth of AgNPs. Nonetheless, the observed trends regarding the variation of standard deviations and morphology, as well as their plausible explanations, should be confirmed in further studies.

## Figures and Tables

**Table 1:** Reagents used for the synthesis of each composite sample.

Sample	AgNO_3_ Weight (mg)	[AgNO_3_] (mg/mL)	CMC Weight (mg)	[CMC] (mg/mL)	CMC/AgNO_3_ Weight Ratio	CMC/Ag Weight Ratio
0.7Ag1	2.41	0.24	300	15.0	124.5	200.0
0.7Ag2	4.82	0.48	300	15.0	62.2	100.0
0.7Ag3	9.45	0.94	300	15.0	31.8	50.0
0.7Ag4	12.59	1.26	300	15.0	23.8	37.5
0.9Ag1	2.41	0.24	300	15.0	124.5	200.0
0.9Ag2	4.82	0.48	300	15.0	62.2	100.0
0.9Ag3	9.45	0.94	300	15.0	31.8	50.0
0.9Ag4	12.59	1.26	300	15.0	23.8	37.5
1.2Ag1	2.41	0.24	300	15.0	124.5	200.0
1.2Ag2	4.82	0.48	300	15.0	62.2	100.0
1.2Ag3	9.45	0.94	300	15.0	31.8	50.0
1.2Ag4	12.59	1.26	300	15.0	23.8	37.5

**Table 2:** Data from particle size distribution obtained for composite samples.

Sample	Mean Particle Size (nm)	Standard Deviation (nm)
0.7Ag1	13.1	5.3
0.7Ag2	26.0	17.7
0.7Ag3	24.6	17.0
0.7 Ag4	10.6	5.1
0.9Ag1	32.6	8.9
0.9Ag2	28.8	14.0
0.9Ag3	28.3	16.4
0.9Ag4	44.1	28.8
1.2Ag1	11.0	4.0
1.2Ag2	20.4	7.1
1.2Ag3	22.3	11.1
1.2 Ag4	19.1	14.8

**Table 3:** Summary of the results obtained from this work.

Characterization Technique	Obtained Results
HAADF-STEM imaging and particle size measuring	The particle size distribution change according to the weight of AgNO_3_ added for the synthesis of nanoparticles, as well as the DS and Mw of CMC used as reducing agent and immobilization medium. The 1.2CMC reagent provides better control on particle size distribution than 0.7CMC and 0.9CMC reagents at the CMC/AgNO_3_ weight ratios used for samples preparation.
BF imaging	The synthesized nanoparticles depict a regular atomic arrangement with an interplanar spacing that is congruent with the family planes {111} of silver.
SAED patterns	The synthesized nanoparticles show diffraction rings related to family planes {111}, {220}, {220} and {311} of the face-centered cubic (FCC) packing of silver.
UV-vis spectroscopy	The UV-Vis spectra obtained from CMC-AgNPs composites show that there are no detectable traces related to Ag^+^ ions, suggesting full reduction of added Ag^+^ to Ag^0^.
ATR-FTIR spectroscopy	The ATR-FTIR spectra recorded from CMC-AgNPs composites suggest an interaction between CMC molecules and AgNPs, that could be attributed to the adsorption of R-CH_2_OCOO^−^ groups onto nanoparticles.
Raman spectroscopy	The Raman spectra obtained from the selected samples confirms the adsorption of CMC chains onto nanoparticles and suggest the formation of a bond between Ag and O in the COO^−^ moieties of CMC.
XPS spectroscopy	The XPS spectra measured from selected composites confirms that the silver in samples is only Ag^0^ and indicates that the AgNPs are immobilized into CMC by coordination bonds. Accordingly, these bonds are attributed to those Ag-O among AgNPs and COO^−^ moieties of CMC. Hence, the key factor for the control of particle size distribution of synthesized AgNPs is the quantity of R-CH_2_OCOO^−^ available for their immobilization. The quantity of these groups varies in direct proportion with the DS of CMC.
Antibacterial activity and cytotoxicity assays	The antibacterial activity and cytotoxicity of the tested samples increase by the use of CMC with higher DS as AgNPs immobilization medium. Therefore, the Ag-O coordination bonds among AgNPs and COO^−^ moieties of CMC modify the ability of nanoparticles to produce and release Ag^+^ into aqueous dispersion, adjusting their antibacterial activity and the induction of cytotoxicity into the tested biological environments.
